# Transmembrane protein 97 exhibits oncogenic properties via enhancing LRP6-mediated Wnt signaling in breast cancer

**DOI:** 10.1038/s41419-021-04211-8

**Published:** 2021-10-06

**Authors:** Huifang Zhu, Zijie Su, Jiong Ning, Liang Zhou, Lifeng Tan, Sapna Sayed, Jiaxing Song, Zhongyuan Wang, Huan Li, Qi Sun, Shanshan Liu, Ou Sha, Feng Leng, Xianxiong Chen, Desheng Lu

**Affiliations:** 1 Guangdong Provincial Key Laboratory of Regional Immunity and Diseases, International Cancer Center, Department of Pharmacology, Shenzhen University Health Science Center, 518055 Shenzhen, China; 2 Department of Research, The Affiliated Tumor Hospital of Guangxi Medical University, 530021 Nanning, China; 3 School of Dentistry, Shenzhen University Health Science Centre, Shenzhen University, 518060 Shenzhen, China; 4 Laboratory of Cancer Biology and Genetics, National Cancer Institute, National Institute of Health, Bethesda, MD 20892-4255 USA

**Keywords:** Protein-protein interaction networks, Breast cancer

## Abstract

Upregulation of transmembrane protein 97 (TMEM97) has been associated with progression and poor outcome in multiple human cancers, including breast cancer. Recent studies suggest that TMEM97 may be involved in the activation of the Wnt/β-catenin pathway. However, the molecular mechanism of TMEM97 action on Wnt/β-catenin signaling is completely unclear. In the current study, TMEM97 was identified as an LRP6-interacting protein. TMEM97 could interact with LRP6 intracellular domain and enhance LRP6-mediated Wnt signaling in a CK1δ/ε-dependent manner. The binding of TMEM97 to LRP6 facilitated the recruitment of CK1δ/ε to LRP6 complex, resulting in LRP6 phosphorylation at Ser 1490 and the stabilization of β-catenin. In breast cancer cells, knockout of TMEM97 attenuated the Wnt/β-catenin signaling cascade via regulating LRP6 phosphorylation, leading to a decrease in the expression of Wnt target genes AXIN2, LEF1, and survivin. TMEM97 deficiency also suppressed cell viability, proliferation, colony formation, migration, invasion, and stemness properties in breast cancer cells. Importantly, TMEM97 knockout suppressed tumor growth through downregulating the Wnt/β-catenin signaling pathway in a breast cancer xenograft model. Taken together, our results revealed that TMEM97 is a positive modulator of canonical Wnt signaling. TMEM97-mediated Wnt signaling is implicated in the tumorigenesis of breast cancer, and its targeted inhibition may be a promising therapeutic strategy for breast cancer.

## Introduction

Breast cancer is one of the most common malignancies diagnosed in women globally. Although the advanced therapeutic approaches have significantly improved the clinical outcome, the recurrence and metastasis remain the leading cause in patients with breast cancer. As a highly complicated disease, breast cancer shows different genetic and phenotypic heterogeneities associated with tumor progression, metastatic potential, clinical outcomes and treatment options [1]. Triple-negative breast cancer (TNBC), negative for estrogen receptor (ER), progesterone receptor (PR), and HER2, is the most aggressive subtype associated with poorer prognosis than other subtypes due to the lack of effective therapeutic targets [2]. Accordingly, there is an urgent need to further understand the molecular mechanism to identify molecular targets for breast cancer therapy.

The Wnt/β-catenin signaling pathway plays a key role in multiple cellular processes involved in embryonic development, stem cell maintenance, and tumorigenesis [3]. This pathway depends on stabilization of β-catenin, which is regulated by a destruction complex composed of casein kinase 1 (CK1), axis inhibitor (AXIN), glycogen synthase kinase-3 (GSK3β), and adenomatous polyposis coli (APC) [4]. Wnt/β-catenin signaling is activated when secreted Wnt ligands bind to their cell membrane receptors frizzled (Fzd) and low-density lipoprotein receptor-related proteins 5/6 (LRP5/6), leading to phosphorylation of LRP5/6 and Dishevelled (DVL), and facilitating the interaction of LRP cytoplasmic tail with AXIN and GSK3β, which prevents the phosphorylation and constitutive degradation of β-catenin. Non-phosphorylated β-catenin accumulates in the cytoplasm and is translocated into the nucleus where it interacts with the T-cell factor/lymphoid enhancer factor 1 (TCF/LEF1) transcription complex to promote transcription of Wnt target genes [3].

LRP6 is a single-pass transmembrane protein with a large extracellular domain (ECD), a transmembrane domain and an intracellular domain which contains a Ser/Thr cluster and five conserved PPPSP motifs [5]. Upon activation by Wnt, LRP6 was phosphorylated at PPPSP motifs by GSK3β and CK1. The phosphorylated PPPSP motifs induce the interaction between LRP6 and the AXIN complex, and directly suppress GSK3β phosphorylation of β-catenin, resulting in the disruption of the β-catenin destruction complex [6]. CK1 family members, including CK1α, CK1γ, CK1δ, and CK1ε, coordinately regulate the response to Wnt stimulus [7]. In the early step of Wnt signaling, CK1ε is constitutively bound to LRP5/6 through its interaction with p120-catenin and E-cadherin or N-cadherin and is activated in response to Wnt3a stimulation [8]. CK1α also interacts with LRP5/6-p120-catenin only after Wnt3a stimulation. CK1ε is required for early responses to Wnt3a, while CK1α participates in the release of p120-catenin from the complex [8]. Canonical Wnt ligands could induce the CK1γ-mediated LRP5/6 phosphorylation at Thr1479 and promote the recruitment of AXIN to the plasma membrane through LRP5/6 [9]. Our recent studies showed that tumor-promoting phorbol ester TPA could stabilize CK1ε, enhance its kinase activity, induce LRP6 phosphorylation at Thr1479 and Ser1490, finally leading to activation of the Wnt/β-catenin pathway [10].

Transmembrane protein 97 (TMEM97), also known as meningioma-associated protein (MAC30) or sigma-2 receptor, is a member of insulin-like growth factor-binding protein family [11]. It is aberrantly upregulated in a variety of cancers, including breast cancer, colorectal cancer, squamous cell lung cancer, esophageal cancer and gastric cancer [12–15]. High expression of TMEM97 was significantly associated with tumor progression, recurrence, and poor survival in breast cancer [13, 16]. In colon cancer, overexpression of TMEM97 was correlated with lymph node metastasis, short survival, and poor prognosis [17]. Knockdown of TMEM97 efficiently inhibited the invasion and EMT in breast cancer cells, accompanying with the reduction of Akt phosphorylation, β-catenin, survivin, and cyclin D1 expressions [18]. Wu et al. reported that downregulation of TMEM97 expression suppressed the proliferation and enhanced apoptosis of gastric cancer cells through reducing the expression of Wnt2, p-GSK-3β, and β-catenin protein in gastric cancer [19]. These studies suggest that TMEM97 may exert its oncogenic effect through activating Wnt/β-catenin and Akt signaling pathways. However, recent studies on the role of TMEM97 in the Wnt/β-catenin pathway are very preliminary, and the molecular mechanism by which TMEM97 activates Wnt/β-catenin signaling is still unclear and needs to be further explored.

In this study, we identified TMEM97 as a novel LRP6-interacting protein. TMEM97 markedly enhanced LRP6-mediated Wnt signaling in a CK1δ/ε-dependent manner. We further investigated the molecular mechanism of TMEM97 in modulating the Wnt/β-catenin pathway. Our results demonstrated that TMEM97 displayed oncogenic effects through regulation of LRP6-mediated Wnt signaling in breast cancer.

## Materials and methods

### Cell culture and reagents

The human embryonic kidney HEK293T cells, normal mammary epithelial Hs578Bst cells and breast cancer Hs578T, MDA-MB-231, MDA-MB-468, MCF7, and BT549 cells were obtained from the American Type Culture Collection (ATCC, Manassas, VA, USA). Breast cancer SW527 cells were provided by Shanghai Sciencelight Biology Science & Technology Co. Ltd (Shanghai, China). HEK293T, Hs578Bst, Hs578T, MCF7, and SW527 cells were grown in Dulbecco’s Modified Eagle’s Medium (DMEM) containing 10% fetal bovine serum (FBS), 1% penicillin-streptomycin in 5% CO_2_ at 37 °C. BT549 cells were cultured in RPMI-1640 medium with 10% FBS. MDA-MB-231 and MDA-MB-468 cells were grown in Leibovitz’s L-15 medium supplemented with 10% FBS and 1% penicillin-streptomycin at 37 °C in a humidified incubator without CO_2_. All antibiotics were purchased from MedChemExpress (MCE, Monmouth Junction, NJ, USA). All chemical reagents were obtained from Sigma-Aldrich (St. Louis, MO, USA) except for special instructions.

### Plasmids

The expression plasmids for C-terminally Flag-tagged and His-tagged TMEM97, PGRMC1 and LRP5 were purchased from WZ Bioscience Inc. The SuperTOPFlash reporter plasmid was provided by Karl Willert, University of California, San Diego. The AP1-Luc and NFAT-Luc reporter vectors, the expression plasmids encoding Wnt1, Wnt3, Fzd5, Fzd7, LRP6, β-catenin, DVL2, and pCMXβgal (β galactosidase, β-gal) have been described previously [10]. The 8×GTIIC-Luc reporter plasmid was purchased from Addgene (Cambridge, MA, USA). For the construction of C-terminally V5-tagged LRP6, CK1δ and CK1ε expression plasmids, the cDNA encoding LRP6, CK1δ, and CK1ε was amplified by RT-PCR and cloned into the pcDNA 3.1/V5-His mammalian expression vector. The N-terminally tagged Flag-CK1ε and Flag-CK1δ expression plasmids were constructed by inserting the corresponding cDNA into the pFlag-CMV2 expression vector. The dominant-negative CK1α, CK1*γ*, CK1δ, and CK1ε, in which a lysine at position 38 was mutated to arginine [20], were generated by site-directed mutagenesis according to the manufacturer’s instructions (Easy Mutagenesis System, TransGen Biotech, Beijing, China). For the construction of His-tagged TMEM97, GST-tagged CK1ε, and GST-tagged LRP6 intracellular fragment (amino acids 1394-1613), the cDNAs encoding human TMEM97, CK1ε, and LRP6 intracellular domain were amplified by PCR. The resulting PCR product of TMEM97 was then subcloned into pET-28 a (+) vector. CK1ε and LRP6 intracellular domain were subcloned into the expression vector pGEX3X. The resulting plasmids were designated pHis-TMEM97, pGST-CK1ε, and pGST-LRP6/1394-1613, respectively. LRP6 mutant lacking the extracellular domain (LRP6-ΔN) was constructed with pFlag-CMV2 expression vector by deleting 1245 amino acids in the N-terminus of LRP6 according to the method described previously [21]. The pEGFP-N1 or pmCherry-N1 vectors were used in constructing the LRP6-GFP or TMEM97-mCherry expression plasmids, respectively. All constructs were verified by DNA sequencing.

### Generation of TMEM97 knockout cell lines by CRISPR-Cas9

TMEM97 was knocked out in Hs578T and MDA-MB-231 cell lines by using CRISPR/Cas9 technology as previously described [22]. Briefly, the single-guide RNA (Table S1) was cloned into the LentiCRISPRv2 vector (Addgene, Cambridge, MA, USA) to obtain the TMEM97 CRISPR/Cas9 KO plasmid. Using the LentiCRISPRv2 vector as a control, Lentivirus was produced by using 10 µg TMEM97 CRISPR/Cas9 KO plasmid or control CRISPR/Cas9 plasmid and two packaging plasmids including 2.5 μg pMD2.G (Addgene, Cambridge, MA, USA) and 7.5 µg psPAX2 (Addgene, Cambridge, MA, USA) in HEK293T cells with 60 µL Lipofectamine 2000 transfection reagent under standard conditions. Media was changed after 12 h of transfection, and the cells were cultured for 2 days. The supernatant was collected and centrifugated at 20,000 rpm for 2 h at 4 °C to harvest virus particles. Virus was immediately added to Hs578T and MDA-MB-231 cells with 8 µg/mL polybrene. After 72 h of infection, cells were then selected for stable expression of cas9 in the presence of 3 µg/mL puromycin (Thermo Fisher Scientific, Waltham, MA, USA) for one week. The puromycin-resistant stable clones were pooled and TMEM97 deficiency was confirmed by Western blotting analysis. Pooled clones and their parental clones were used to examine their biological behaviors.

In order to verify stable TMEM97 gene knockout in MDA-MB-231 cells, the target sites were amplified from the extracted DNA of CRISPR-transduced MDA-MB-231 cells, and amplicons were sequenced. The genomic DNA sequences spanning the target site in parental and TMEM97 knockout MDA-MB-231 cells were presented in Fig. S1 (Fig. S1).

### Luciferase reporter gene assays

HEK293T cells were transfected in 24-well plates with the SuperTOPFlash reporter plasmid, control plasmid for β-gal and the indicated amounts of expression plasmids using Lipofectamine 2000 according to the manufacturer’s instructions. Luciferase assays were conducted using a luciferase assay kit (Promega, Shanghai, China). The luciferase values were normalized using a β-gal internal control to determine the variation in transfection efficiency.

### Mass spectrometry assays

The mass spectrometry assay was performed as described previously [23]. Briefly, HEK293T cells were transfected with N-terminally Flag-tagged LRP6-ΔN expression vector or empty vector. The total protein of the cells was harvested with RIPA buffer containing 50 mM Tris-HCl at pH 7.4, 150 mM NaCl, 1% Nonidet P-40, 0.1% SDS, 0.5% sodium deoxycholate, 1 mM EDTA, 1 mM PMSF, protease inhibitors (Bimake, Beijing, China), and phosphatase inhibitors (Topscience, Shanghai, China), followed by centrifugation. Immunoprecipitations were performed using anti-Flag agarose beads (Bimake, Beijing, China) incubated with the supernatant overnight at 4 °C. The beads were washed three times with RIPA buffer. Beads with extracted proteins were digested by trypsin (Promega, Shanghai, China). The peptide sequences from these proteins were then extracted for mass spectrometry analysis on a Q Exactive mass spectrometer (Thermo Fisher Scientific, San Jose, CA, USA).

### Quantitative real-time PCR analyses

Total RNA was extracted by RNAiso Plus (TaKaRa, Beijing, China) and subsequently reverse-transcribed into cDNA using the Primescript RT Reagent Kit (TaKaRa, Beijing, China) according to the manufacturer’s instructions. Quantitative PCR analysis was carried out with 2× SYBR Green qPCR Master Mix (Promega, Shanghai, China). The primer sequences are listed in Table S1.

### Immunoblot analyses

Cells or tumor tissues were lysed in RIPA buffer, followed by sonication. The lysates were quantified by a bicinchoninic acid (BCA) protein assay kit (Beyotime, Shanghai, China). Equal amounts of protein samples were separated by sodium dodecyl sulfate-polyacrylamide gel electrophoresis (SDS-PAGE) and transferred to poly-vinylidenefluoride (PVDF) membranes. Western blotting was performed with the indicated primary antibodies at 4 °C overnight. Then the PVDF membranes were incubated with secondary antibodies for 1 h at room temperature. After incubation with ECL Plus Western Blotting Substrate (Thermo Fisher Scientific, Shanghai, China), the immunoblots were developed by the Tanon 5200 Chemiluminescent Imaging System (Tanon Science and Technology, Shanghai, China). The primary and secondary antibodies are given in Table S2.

### Co-immunoprecipitation assays

Total cell lysates were harvested in 500 µL RIPA buffer with freshly added protease inhibitor and phosphatase inhibitor cocktails, followed by centrifugation at 12,000 rpm for 15 min at 4 °C. The lysates were quantified and incubated with respective antibodies and protein G beads at 4 °C overnight. Immunoprecipitations were conducted using anti-Flag or anti-V5 agarose beads (Bimake, Beijing, China), respectively. The beads were washed four times with RIPA buffer. Proteins were eluted by boiling the samples in SDS loading buffer and analyzed by SDS-PAGE and immunoblotting.

### GST pull-down assays

His-TMEM97, GST, and GST-LRP6/1394-1613 fusion proteins were expressed in *Escherichia coli (E. coli)* BL21 and the expression of fusion proteins was induced with IPTG (Sigma-Aldrich, Shanghai, China) at 16 or 28 °C for 8–12 h. His-TMEM97 was purified by NiNTA Purification System while GST and GST-fusion proteins were purified by affinity chromatography with glutathione-sepharose 4B beads (GE Healthcare, Piscataway, NJ, USA). For the in vitro pull-down assay, His-TMEM97 fusion protein was incubated with GST or GST-LRP6/1394-1613 fusion proteins at 4 °C rotating for 4 h, then pulled down with glutathione-sepharose 4B beads. The immunoprecipitates were washed five times with PBS buffer. The beads were boiled in 95 °C with SDS loading buffer and analyzed by SDS-PAGE and immunoblotting.

### Confocal imaging assays

HEK293T cells cultured on coverslips were transfected with TMEM97-mCherry expression plasmid alone or in combination with LRP6-GFP expression vector. After 48 h of transfection, cells were fixed with 4% paraformaldehyde at room temperature for 15 min, the cell nucleus was stained with DAPI (4,6-diamidino-2-phenylindole) and cells were mounted. The slides were observed with a fluorescence microscope (LSM880, ZEISS, Germany) at the Instrumental Analysis Center of Shenzhen University.

### Cell viability assays

TMEM97 knockout Hs578T and MDA-MB-231 cells and their parental wild-type counterparts were seeded at 1×10^3^ cells/well in 96-well plates. MTT reagent (Sangon Biotech, Shanghai, China) (5 mg/mL, 20 µL/well) was added after 24 h, 48 h, and 72 h of culture, and then incubated for another 4 h. The formazan crystals were dissolved in 100 µL DMSO, and the absorbance of the formazan solution was detected at 570 nm.

### BrdU cell proliferation assays

TMEM97 knockout Hs578T and MDA-MB-231 cells and their parental wild-type counterparts were plated at 3×10^3^ cells per well in 96-well plates, respectively. The BrdU incorporation assay was conducted using the Cell Proliferation ELISA BrdU Colorimetric Kit (Roche, Shanghai, China) according to the manufacturer’s instructions. Each group was performed in five replicates.

### Colony formation assays

TMEM97 knockout Hs578T and MDA-MB-231 cells and their parental wild-type counterparts were seeded in a 6-well plate with a ratio of 500 cells per well. When the colonies were obviously visible, cells were washed twice with PBS, fixed with 4 % paraformaldehyde for 15 min, stained with crystal violet and photomicrography. To conduct quantitative analysis, colonies with a diameter larger than 30 µm were counted with ImageJ software. The experiments were replicated three times.

### In vitro migration and invasion analyses

As described previously [24], TMEM97 knockout Hs578T and MDA-MB-231 cells and their parental wild-type counterparts were seeded in 24-transwell chambers with 8 µm pore membrane in serum-free medium with 1×10^5^ cells. The lower chamber contained medium with 20% FBS. After incubation for 18–36 h, the cells were fixed with 4% paraformaldehyde for 15 min and then rinsed with PBS, followed by staining with 0.1% crystal violet. The unmigrated cells on the upper side of the membrane were rubbed away, and the migrated cells were photographed. Invasion assay was same as migration assay, except that the transwell chambers were precoated with Matrigel (Corning life science, Corning, NY, USA). To conduct quantitative analysis, the cells were eluted with 33% acetic acid and the absorbance was measured at 570 nm.

### Sphere formation assays

TMEM97 knockout Hs578T and MDA-MB-231 cells and their parental wild-type counterparts were seeded at 500 cells per well in a 24-well plate with an ultra-low attachment surface containing DMEM/F12 medium (2% B-27, 10 ng/mL EGF, 10 ng/mL FGF, and 10 µg/mL insulin). After 10 days of culture, spheres with a diameter larger than 30 µm were counted, and representative areas were photographed using a light microscope. ImageJ software was used to analyze statistically the sphere numbers. The experiments were replicated three times.

### Animal model study

All animal experiments were conducted by the protocols with the permission of Animal Research of Shenzhen University (permit number AEWC-201412003). For the breast cancer xenograft animal model, female BALB/c nude mice (6-week old) were purchased from Beijing Vital River Laboratory Animal Technology Company (Beijing, China). TMEM97 knockout MDA-MB-231 cells and the parental counterparts were implanted s.c. into the right flank of nude mice at a dosage of 1×10^7^ cells per mouse. Following implantation, tumor growth was closely observed. Tumor volumes were measured with a caliper twice a week and calculated using the following formula: 0.528 × (length/2) × (width/2) [2]. CO_2_ euthanasia was performed when tumor size reached 10 mm in diameter. The tumors were excised, weighed and fixed in formalin for histological analysis. Mouse experiments were performed without knowledge of subject’s genotype.

### Histological analyses

The tumors were fixed in formalin, embedded in paraffin, and sectioned. Hematoxylin and eosin (HE) staining and immunohistochemistry analysis were conducted as described previously [24]. The primary antibodies used are listed in Table S2.

### Statistical analyses

Statistical analyses were performed using GraphPad prism 7.0 software (GraphPad, RRID: SCR_000306). Student’s *t* test was applied to compare the difference between two groups when the data showed a normal distribution. One-way analysis of variance (ANOVA) with Dunn’s multiple comparisons test was carried out to compare the means of several groups. The variance was similar between the groups that are being statistically compared. Data were presented as mean ± SD. Differences with *P* value <0.05 were considered statistically significant. All the experiments were replicated at least three times.

## Results

### TMEM97 is identified as an interacting partner of LRP6

In order to identify downstream factors of LRP6 in the transduction of Wnt signaling, HEK293T cells were transiently transfected with an expression vector for Flag-tagged LRP6 mutant comprising the transmembrane and cytoplasmic domain, but lacking the extracellular domain. Flag-tagged LRP6 mutant was pulled down by anti-Flag M2 agarose, followed by mass spectrometry to identify the potential interacting partners of LRP6 mutant. As expected, several known LRP6-interacting proteins such as CK1α, CK1ε, GSK3β, and MACF1 were detected by mass spectrometry analysis [6, 25, 26]. Surprisingly, the results from mass spectrometry analysis revealed that anti-Flag M2 agarose could pull down TMEM97 protein, suggesting that LRP6 may interact with TMEM97.

**Fig. 1:**
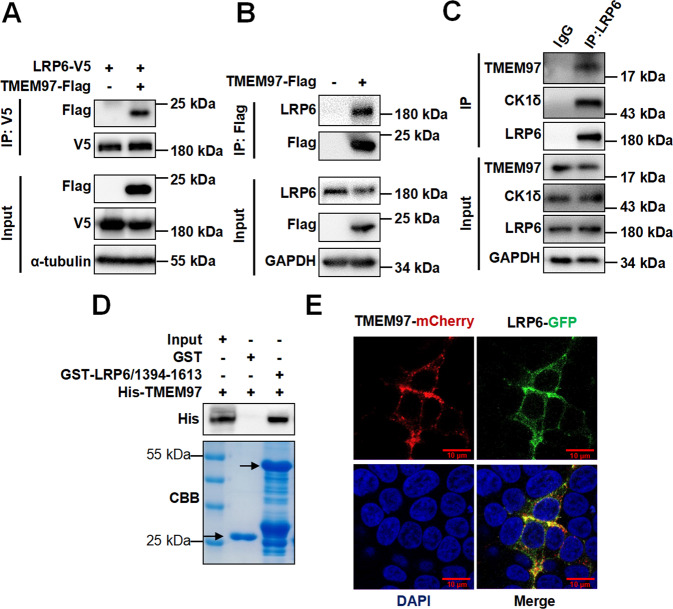
TMEM97 directly interacts with LRP6. **A** HEK293T cells were cotransfected with LRP6-V5 and TMEM97-Flag expression plasmids. The lysates were immunoprecipitated with anti-V5 beads. Immunoblotting was performed using the indicated antibodies. **B** The expression vector for TMEM97-Flag was transfected into HEK293T cells and immunoprecipitation was performed using anti-Flag beads. Immunoblotting was performed by using the indicated antibodies. **C** Immunoprecipitation of HEK293T cell lysates was performed using control IgG or anti-LRP6 antibody. Immunoblotting was carried out using the indicated antibodies. The images shown are representative of data generated in at least three independent experiments. **D** The in vitro interaction between TMEM97 and LRP6 was detected by a GST pull-down assay. Purified His-TMEM97 fusion protein was incubated with GST and GST-LRP6/1394-1613 fusion protein. Fusion protein-bound glutathione-agarose beads were analyzed by immunoblotting with anti-His antibody and the corresponding gels were stained with Coomassie brilliant blue (CBB). The arrows indicate proteins with correct molecular masses (bottom panel). **E** Representative images of LRP6-GFP and TMEM97-mCherry colocalization. Scale bar = 10 μm. HEK293T cells were transfected with TMEM97-mCherry and LRP6-GFP plasmids. The cells were fixed and stained with DAPI. The colocalization of LRP6-GFP and TMEM97-mCherry was detected using a ZEISS-LSM880 laser scanning confocal microscope.

TMEM97 is a transmembrane protein with a molecular weight of 18-21.5 kDa, which is involved in multiple cancers and cholesterol homeostasis [27]. To confirm the interaction of TMEM97 with LRP6, an expression plasmid for TMEM97-Flag was transfected into HEK293T cells with or without LRP6-V5 expression vector. The anti-V5 beads or anti-Flag beads were used for the immunoaffinity purification. As shown in Fig. 1A-B, Flag-TMEM97 was immunoprecipitated using anti-V5 beads from the lysate of HEK293T cells transfected with Flag-TMEM97 and LRP6-V5 expression vectors (Fig. 1A). The endogenous LRP6 protein was also immunoprecipitated by Flag-TMEM97 (Fig. 1B). Apart from these observations, endogenous interaction between LRP6 and TMEM97 was detected by co-immunoprecipitation using an antibody against LRP6 in HEK293T cells (Fig. 1C). These results clearly showed that TMEM97 and LRP6 coexisted in the same protein complex in HEK293T cells. Moreover, a GST pull‐down assay was performed to examine the interaction of these two proteins. The expression plasmids for GST-tagged intracellular domain of LRP6 (GST-LRP6/1394-1613) and His-tagged TMEM97 (His-TMEM97) were constructed. The GST-fusion protein was expressed in *E. coli* and purified by glutathione-sepharose beads. The glutathione-sepharose-bound GST-fusion protein was incubated with purified His-TMEM97. Our results showed that His-TMEM97 interacted with GST-LRP6/1394-1613 (Fig. 1D), suggesting that TMEM97 may interact with the intracellular domain of LRP6. Consistent with this, His-TMEM97 also interacted with GST-tagged LRP6 mutant lacking the extracellular domain (GST-LRP6ΔN) (data not shown).

To assess the intracellular localization of TMEM97, a recombinant plasmid expressing TMEM97-mCherry fusion protein was constructed and expressed in HEK293T cells. This fusion protein was detected in the cell membrane and cytoplasm. Partial colocalization of TMEM97-mCherry with LRP6-GFP was observed in cotransfected HEK293T cells, indicating an interaction between the two proteins (Fig. 1E).

### TMEM97 specifically enhances LRP6-mediated Wnt signaling via regulating LRP6 phosphorylation

**Fig. 2:**
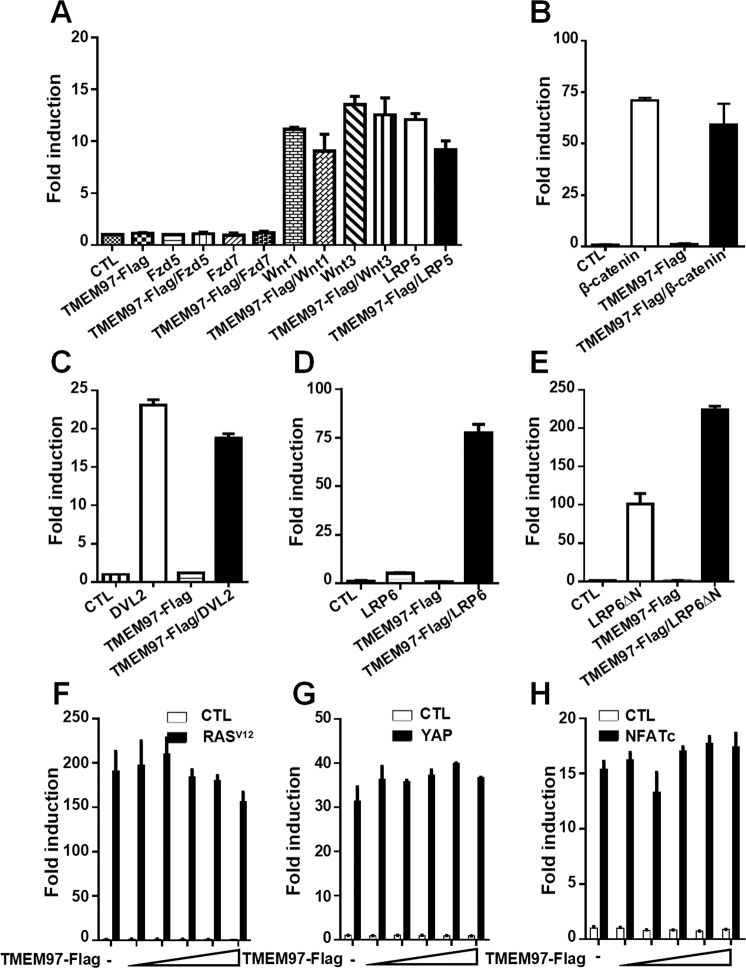
TMEM97 specifically promotes LRP6-mediated Wnt signaling. **A**–**E** The SuperTOPFlash reporter gene was transfected into HEK293T cells together with either TMEM97 expression vector alone or combined with expression plasmids encoding Wnt1, Wnt3, Fzd5, Fzd7, or LRP5 (A). **B**–**E** Similar to panel (**A**) except that expression plasmids encoding β-catenin (**B**), DVL2 (**C**), LRP6 (**D**), and LRP6-ΔN (**E**) were used. **F** HEK293T cells were transfected with an AP1-Luc reporter and empty vector or a constitutively active Ras^V12^ expression plasmid. **G** HEK293T cells were transfected with an 8×GTIIC-Luc reporter and empty vector or an expression plasmid for YAP. **H** HEK293T cells were transfected with an NFAT-Luc reporter and empty vector or an expression plasmid for NFATc. The luciferase values were normalized to β-gal activities.

We explored the effects of TMEM97 on the Wnt/β-catenin signaling pathway using the SuperTOPFlash reporter, which contains eight TCF/LEF-binding sites in the promoter of a firefly luciferase reporter gene. Induction of Wnt/β-catenin-dependent gene expression can be detected by monitoring luciferase activity of the SuperTOPFlash reporter gene [28, 29]. The SuperTOPFlash reporter was transfected into HEK293T cells along with either TMEM97 expression vector alone or combined with Wnt1, Wnt3, Fzd5, Fzd7, LRP5, LRP6, constitutively active LRP6 lacking the extracellular domain (LRP6ΔN), DVL2, and β-catenin expression plasmids, respectively. Overexpression of TMEM97 had little effect on the activity of SuperTOPFlash reporter activated by Wnt1, Wnt3, Fzd5, Fzd7, LRP5, DVL2, and β-catenin (Fig. 2A–C). Surprisingly, TMEM97 dramatically enhanced the transcriptional activity stimulated by either wild-type LRP6 or LRP6ΔN (Fig. 2D, E), whereas it was unable to influence the luciferase activities of AP1-Luc, NFAT-Luc, and Hippo reporter (8×GTIIC-Luc) (Fig. 2F–H). Collectively, these results indicate that TMEM97 could specifically promote LRP6-mediated Wnt signaling in an LRP6 extracellular domain-independent manner.

**Fig. 3:**
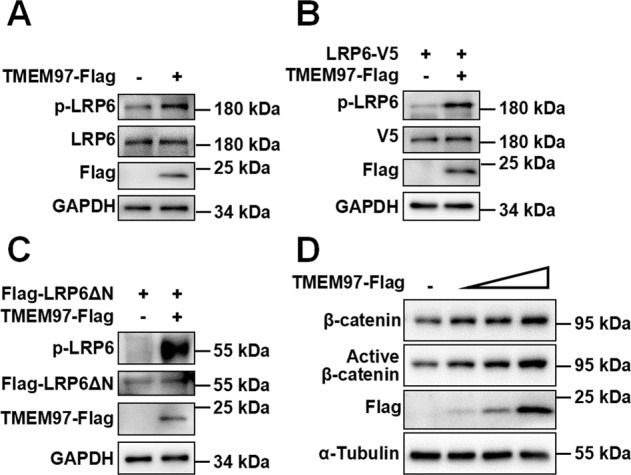
TMEM97 increases LRP6 phosphorylation at Ser1490 and enhances the protein level of β-catenin. **A** TMEM97 enhanced endogenous LRP6 phosphorylation at Ser1490. HEK293T cells were transfected with TMEM97-Flag expression vector. The phosphorylated LRP6, total LRP6, and TMEM97-Flag were detected by immunoblotting. **B**–**C** TMEM97 increased the phosphorylation of exogenous LRP6 (**B**) and LRP6-ΔN (**C**). HEK293T cells were transfected with an expression vector for LRP6-V5 alone or combined with TMEM97-Flag expression vector. Immunoblotting was performed using the indicated antibodies. **D** TMEM97 enhanced the level of active β-catenin and total β-catenin. HEK293T cells were transfected with increasing amounts of TMEM97 expression plasmid (0, 100, 200, and 400 ng). Total β-catenin and active β-catenin were detected by immunoblotting. The images shown are representative of data generated in at least three independent experiments.

A crucial step of the Wnt signaling cascade is the phosphorylation of LRP6, which is important for β-catenin stabilization and the activation of Wnt signaling [30]. We next examined whether TMEM97 has any effect on LRP6 phosphorylation. HEK293T cells were transfected with TMEM97 expression vector with or without an expression plasmid for LRP6. Western blot analysis illustrated that overexpression of TMEM97 significantly increased the phosphorylation levels of endogenous and exogenous LRP6 at Ser1490 (Fig. 3A, B). TMEM97 also increased the phosphorylation of LRP6ΔN (Fig. 3C). Importantly, TMEM97 could upregulate the expression of active β-catenin and total β-catenin in a dose-dependent manner (Fig. 3D). Together, these results suggest that TMEM97 may potentiate LRP6-mediated Wnt signaling via the regulation of LRP6 phosphorylation.

### TMEM97 enhances LRP6-mediated Wnt signaling in a CK1δ/ε-dependent manner

**Fig. 4:**
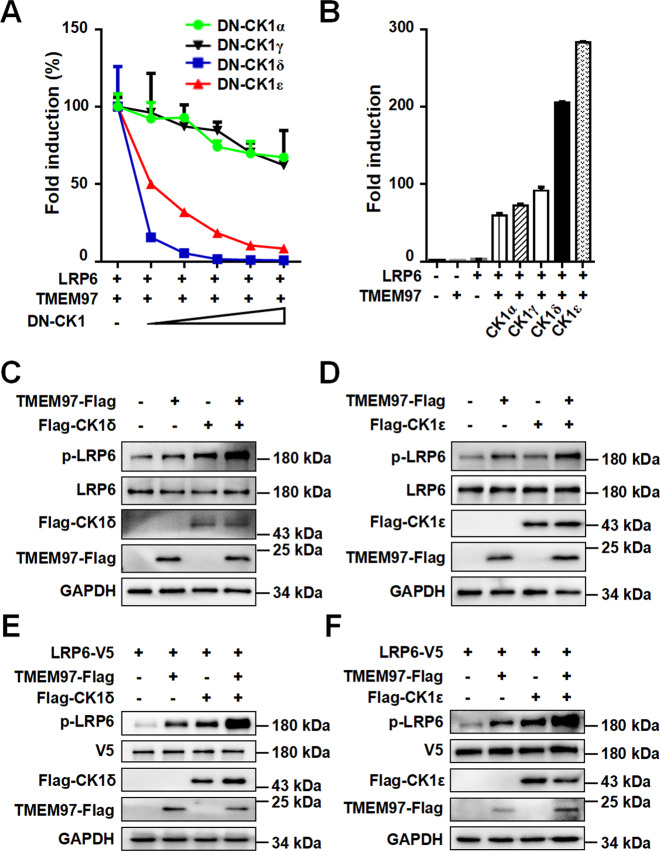
TMEM97 enhances LRP6-mediated Wnt signaling in a CK1δ/ε-dependent manner. **A** The SuperTOPFlash reporter gene was transfected into HEK293T cells together with expression vectors for TMEM97 and LRP6 in the absence or presence of increasing amounts of dominant negative (DN) CK1α, CK1γ, CK1δ, and CK1ε expression plasmids (0, 25, 50, 100, and 200 ng), respectively. **B** The SuperTOPFlash reporter gene was transfected into HEK293T cells together with expression vectors for TMEM97 and LRP6 with or without CK1α, CK1γ, CK1δ, and CK1ε expression plasmids, respectively. **C**–**D** TMEM97 promoted LRP6 phosphorylation via CK1δ/ε. HEK293T cells were transfected with an expression vector for TMEM97-Flag together with Flag-CK1δ (**C**) or Flag-CK1ε (**D**) expression plasmids as indicated. Phosphorylated LRP6 and total LRP6 were detected by immunoblotting. **E**–**F** HEK293T cells were transfected with the expression plasmids for LRP6 and TMEM97 in the absence or presence of Flag-CK1δ (**E**) or Flag-CK1ε (**F**) expression plasmids as indicated. Phosphorylated LRP6 and total LRP6 were detected by immunoblotting. The images shown are representative of data generated in at least three independent experiments.

Concerning the important role of CK1 in the phosphorylation of LRP6, we tested whether CK1 is implicated in TMEM97-induced LRP6 signaling. The SuperTOPFlash reporter was transfected into HEK293T cells along with the expression plasmids for TMEM97 and LRP6 in the absence or presence of increasing amounts of dominant negative mutants of CK1α, CK1γ, CK1δ, and CK1ε, respectively. As shown in Fig. 4A, the dominant negative mutants of CK1δ/ε markedly attenuated TMEM97/LRP6-mediated transcription of the reporter gene, while the dominant negative CK1α (DN-CK1α) and DN-CK1γ exerted a much weaker inhibitory effect on TMEM97/LRP6-mediated signaling. Conversely, overexpression of either CK1δ or CK1ε further enhanced TMEM97/LRP6-mediated signaling, but the presence of CK1α or CK1γ had little effect on TMEM97/LRP6-induced Wnt pathway activation. Moreover, the expression of exogenous TMEM97 potentiated CK1δ/ε-induced phosphorylation of endogenous and exogenous LRP6 at Ser1490 in HEK293T cells (Fig. 4C–F).

### TMEM97 promotes the association of LRP6 with CK1δ/ε

**Fig. 5:**
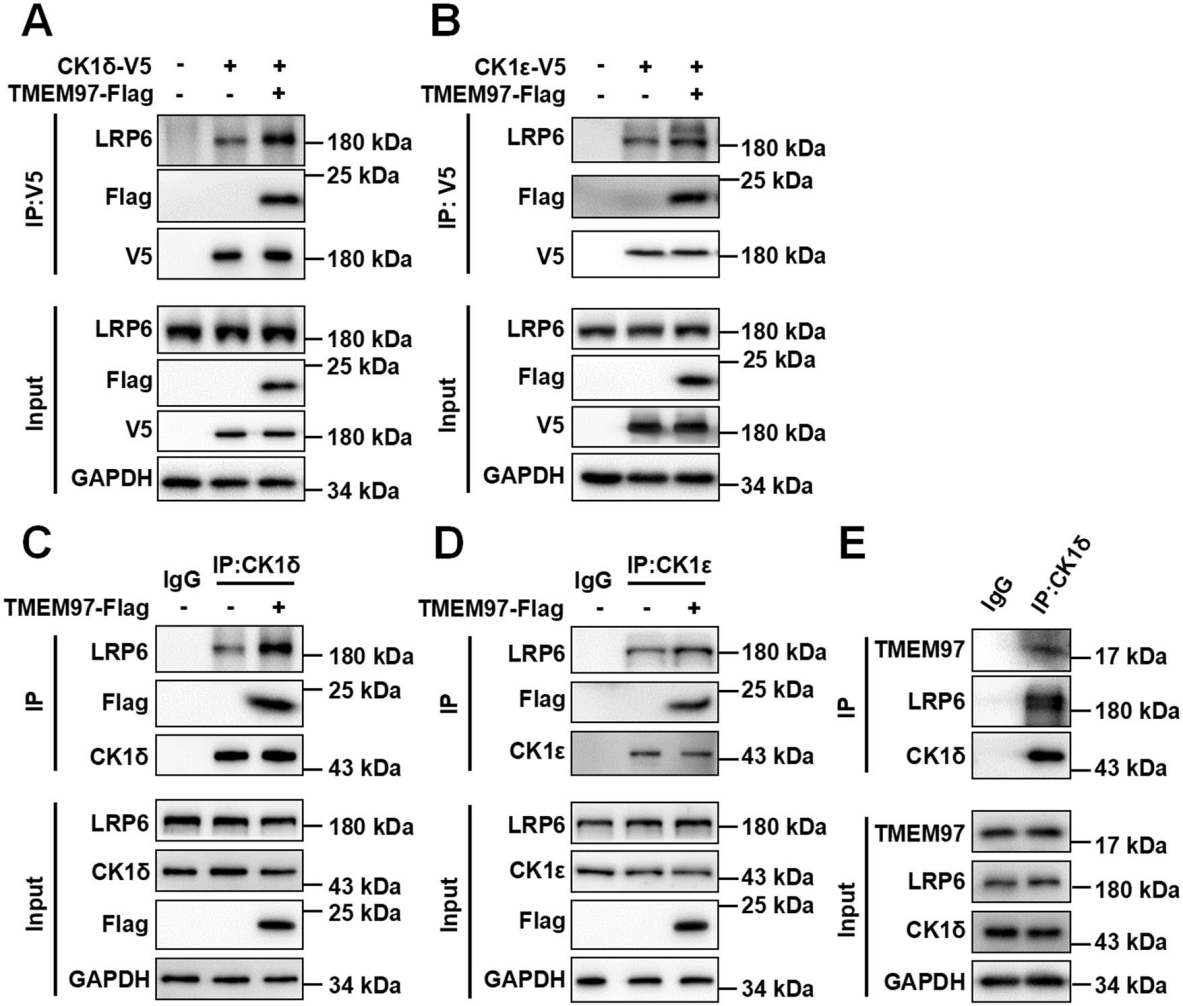
TMEM97 potentiates the interaction of LRP6 with CK1δ/ε. **A** HEK293T cells were transfected with the vectors expressing CK1δ-V5 (A) or CK1ε-V5 (**B**) alone or combined with TMEM97-Flag expression vector. Cell lysates were immunoprecipitated with anti-V5 beads. Immunoblotting was carried out using the indicated antibodies. **C**–**D** HEK293T cells were transfected with TMEM97-Flag expression plasmid. Cell lysates were immunoprecipitated with IgG or anti-CK1δ (**C**) or anti-CK1ε (**D**) antibodies. Immunoblotting was performed using the indicated antibodies. **E** Cell lysates from MDA-MB-231 cells were subjected to immunoprecipitation with IgG or anti-CK1δ antibody. Immunoblotting was performed using the indicated antibodies. The images shown are representative of data generated in at least three independent experiments.

To determine the effect of TMEM97 on the interaction of LRP6 with CK1, a co-immunoprecipitation assay was performed using HEK293T cells that were transiently transfected with either TMEM97 expression vector alone or combined with plasmids expressing CK1δ-V5 or CK1ε-V5. The results showed that the presence of TMEM97 increased the association of endogenous LRP6 with exogenous CK1δ or CK1ε (Fig. 5A, B). We also observed a stronger endogenous interaction between LRP6 and CK1δ or CK1ε in HEK293T cells transfected with TMEM97 expression vector (Fig. 5C, D). Moreover, an anti-CK1δ antibody pulled down endogenous LRP6 and TMEM97 in unmanipulated breast cancer MDA-MB-231 cells (Fig. 5E), demonstrating the existence of TMEM97–LRP6-CK1 complex.

### Knockout of TMEM97 attenuates the Wnt/β-catenin signaling cascade via regulating LRP6 phosphorylation in breast cancer cells

**Fig. 6:**
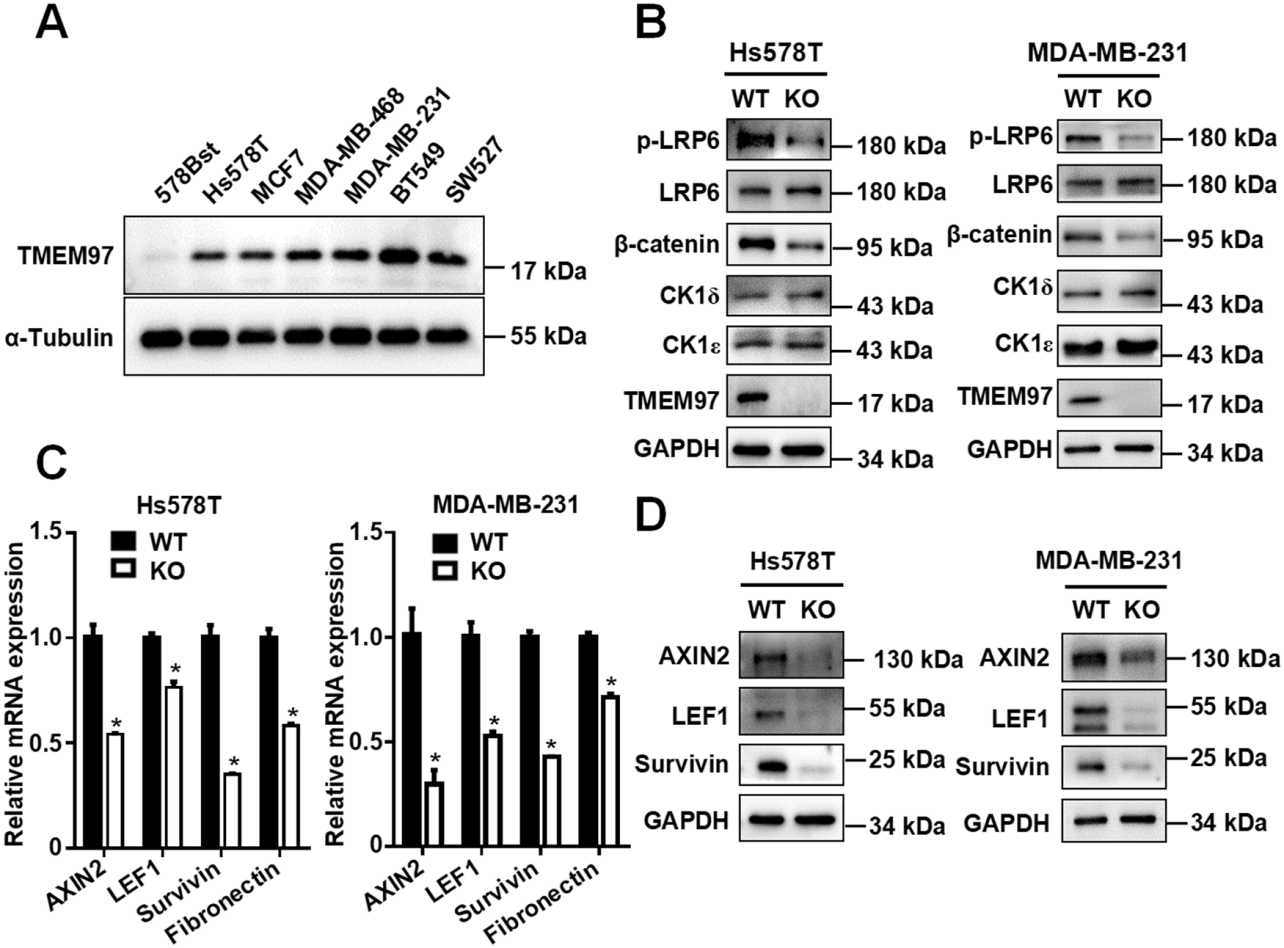
TMEM97 knockout attenuates the Wnt/β-catenin signaling cascade through regulating LRP6 phosphorylation at Ser1490 in breast cancer cells. **A** The expression of TMEM97 protein was detected by immunoblot analysis in normal mammary epithelial Hs578Bst cells and breast cancer Hs578T, MCF-7, MDA-MB-468, MDA-MB-231, BT549, and SW527 cells. **B** Knockout of TMEM97 downregulated the Wnt/β-catenin pathway via reducing LRP6 phosphorylation at Ser1490 in Hs578T and MDA-MB-231 cells. **C** Knockout of TMEM97 decreased the mRNA expression of Wnt target genes AXIN2, LEF1, survivin, and fibronectin in Hs578T and MDA-MB-231 cells. Data represent the mean ± SD (*n* = 3, *P < 0.05). **D** Western blot analysis of the protein expression of Wnt target genes AXIN2, LEF1, and survivin in Hs578T and MDA-MB-231 cells with TMEM97 knockout. The images shown are representative of data generated in at least three independent experiments.

Previous studies have revealed that TMEM97 is highly expressed in breast cancer, and associated with disease progression and poor prognosis [13]. We checked the expression of TMEM97 in six breast cancer cell lines (SW527, BT549, MDA-MB-231, MDA-MB-468, MCF7, and Hs578T) and one normal mammary epithelial cell line (Hs578Bst) by Western blot. The results showed that the expression of TMEM97 protein was upregulated in breast cancer cell lines compared to normal mammary epithelial cells (Fig. 6A). To assess the potential role of TMEM97 in breast cancer pathogenesis, TMEM97 was knocked out in Hs578T and MDA-MB-231 cells using the CRISPR-Cas9 system. The knockout cells were validated by Western blot analysis (Fig. 6B). Compared to the wild-type cell lines, the levels of phosphorylated LRP6 and total β-catenin were markedly reduced in Hs578T and MDA-MB-231 cells with TMEM97 deficiency, while TMEM97 knockout had little effect on the expression of total LRP6, CK1ε, and CK1δ (Fig. 6B). Furthermore, knockout of TMEM97 downregulated the expression of Wnt target genes AXIN2, LEF1, and survivin at both the mRNA and protein level in Hs578T and MDA-MB-231cells (Fig. 6C, D). Additionally, TMEM97 knockout MDA-MB-231 cells were infected with lentivirus harboring TMEM97, resulting in re-expression of TMEM97 in the knockout cells. We observed that the effect of TMEM97 knockout on Wnt/β-catenin signaling was reversed upon reintroduction of TMEM97 in MDA-MB-231 cells (Fig. S2). Taken together, these results indicate that TMEM97 could promote the LRP6-mediated Wnt signaling pathway via regulating LRP6 phosphorylation in breast cancer cells.

### TMEM97 deficiency suppresses cell viability, proliferation, colony formation, migration and invasion in breast cancer cells

**Fig. 7:**
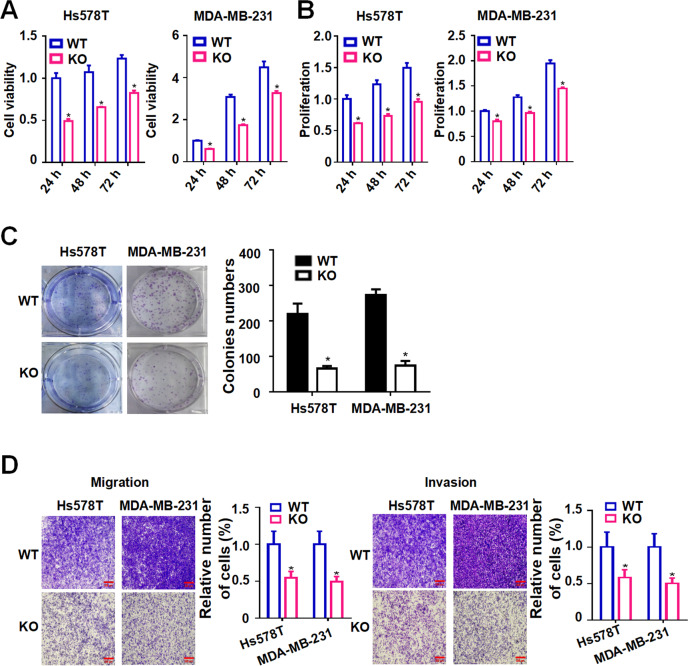
TMEM97 knockout reduces cell viability, proliferation, colony formation, migration and invasion in breast cancer cells. TMEM97 knockout Hs578T and MDA-MB-231 cells (KO) and their parental counterparts (WT) were seeded at 1×10^3^ cells/well in 96-well plates. After 24 h, 48 h, and 72 h of culture, MTT assay was used to detect cell viability (**A**). Cell proliferation was detected using BrdU incorporation assay (**B**). **C** Colony formation of TMEM97 knockout Hs578T and MDA-MB-231 cells (KO) and their parental counterparts (WT). The right panel is the diagrammatic representation of quantitative data which showed the relative number of colonies formed in knockout cells and their parental counterparts at day 10. The number of colonies was calculated by the ImageJ software (*n* = 3). (D) Knockout of TMEM97 blocked the migration and invasion of Hs578T and MDA-MB-231cells. The right panel is the quantitative data on the relative number of migrated or invaded cells in the graphs (*n* = 3). Scale bar = 500 μm. Data were shown as mean ± SD. *P < 0.05 compared with vehicle control.

We next investigated the effect of TMEM97 knockout on the biological behaviors of breast cancer cells (Hs578T and MDA-MB-231). Breast cancer cells with TMEM97 knockout displayed a notable decrease in cell viability and proliferation compared with parental Hs578T and MDA-MB-231 cells (Fig. 7A, B). Using a colony formation assay, we showed that deficiency of TMEM97 reduced the capability of colony formation in Hs578T and MDA-MB-231 cells (Fig. 7C). The migration of breast cancer cells was measured by transwell assay. Our results revealed that the migratory ability was suppressed in Hs578T and MDA-MB-231 cells with TMEM97 knockout (Fig. 7D). Moreover, TMEM97 deficiency caused an obvious decrease in the numbers of penetrated Hs578T and MDA-MB-231 cells in the transwell assays using Matrigel-coated chambers (Fig. 7D). In addition, TMEM97 reintroduction rescued the effect of TMEM97 deficiency on cell viability, proliferation, colony formation, migration and invasion in MDA-MB-231 cells (Fig. S3). Collectively, our results suggest that TMEM97 may play an important role in cell viability, proliferation, colony formation, migration, and invasion in breast cancer cells.

### Knockout of TMEM97 inhibits the stemness properties of breast cancer cells

Since Wnt/β-catenin signaling has been implicated in stem cell-like properties in breast cancer [31], we examined the effect of TMEM97 knockout on the stemness of breast cancer cells. The self-renewal capacity was analyzed by the sphere formation assay. The expression of stemness marker genes LGR5, Sox2, and Slug was detected by real-time PCR and Western blot analysis. As revealed by the sphere formation assay, knockout of TMEM97 markedly decreased the number and size of tumor spheres in Hs578T and MDA-MB-231 cells (Fig. S4A). Consistently, the expression of LGR5, Sox2, and Slug was significantly downregulated at both the mRNA and protein levels in TMEM97-deficient Hs578T and MDA-MB-231 cells (Fig. S4B, C).

### Knockout of TMEM97 downregulates the Wnt/β-catenin signaling pathway and inhibits breast tumor growth

**Fig. 8:**
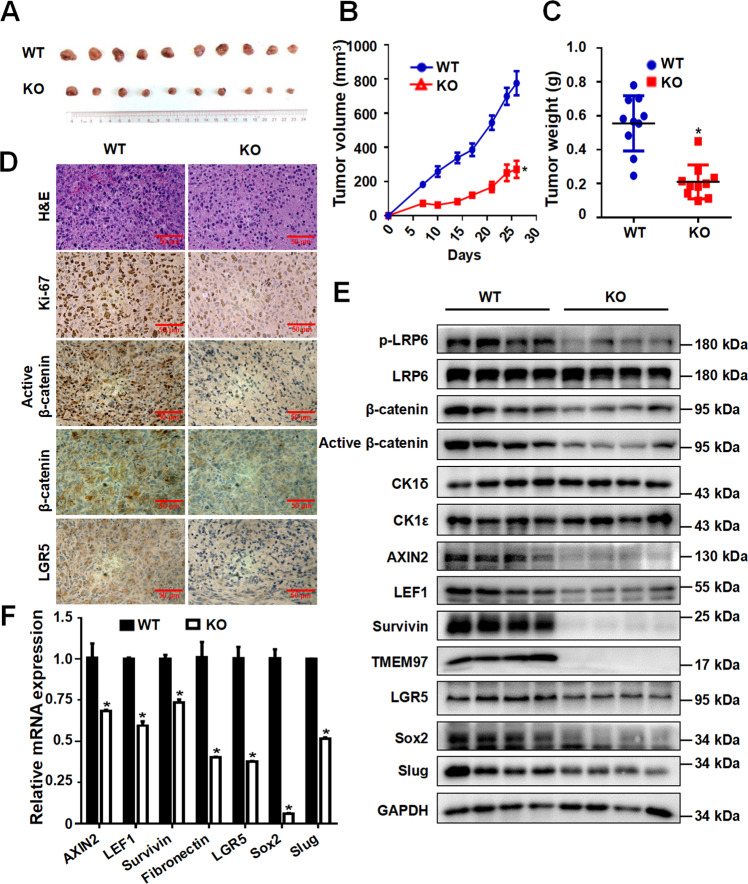
TMEM97 Knockout inhibits tumor growth in vivo through downregulating Wnt/β-catenin signaling in MDA-MB-231 cell xenografts. **A** Images of tumors from TMEM97 knockout group (KO) and the control group (WT). **B** Tumor volume. **C** Tumor weight. Each dot represents an individual mouse. **D** H&E staining, Ki-67 antibody staining, active β-catenin antibody staining, total β-catenin antibody staining, and LGR5 antibody staining. Scale bar = 50 μm. **E** The protein expression levels of phosphorylated LRP6, total LRP6, active β-catenin, total β-catenin, CK1δ, CK1ε, AXIN2, LEF1, survivin, TMEM97, LGR5, Sox2, and Slug in tumor samples were detected by immunoblotting. The images shown are representative of data generated in at least three independent experiments. **F** The mRNA expression levels of AXIN2, LEF1, survivin, fibronectin, LGR5, Sox2, and Slug were quantitated by real-time PCR. Data represent the mean ± SD (*n* = 3, *P < 0.05).

To examine the role of TMEM97 in breast cancer tumorigenesis, TMEM97-deficient MDA-MB-231 cells and parental counterparts were subcutaneously injected into nude mice to generate a xenograft tumor model. After implantation for 28 days, tumor growth was significantly suppressed in the TMEM97-deficient cell group. Knockout of TMEM97 resulted in an obvious reduction in the volume and weight of tumor xenografts compared with the control group (Fig. 8A–C). Histological analysis of tumor samples showed that tumor cell density and Ki67 expression were decreased in tumor tissues from TMEM97 knockout group (Fig. 8D).

Immunohistochemical staining revealed that tumor tissues from TMEM97-deficient group displayed decreased expression of active and total β-catenin (Fig. 8D). Impressively, knockout of TMEM97 reduced the expression of stem cell marker LGR5 (Fig. 8D). Moreover, we observed a marked decrease in the levels of phosphorylated LRP6, active and total β-catenin, Wnt target genes (AXIN2, LEF1, and survivin) and stemness marker genes (LGR5, Sox2, and Slug), as detected by Western blot analysis (Fig. 8E). Real-time PCR also showed significantly reduced mRNA expression of the Wnt target genes (AXIN2, LEF1, and survivin) and stemness marker genes (LGR5, Sox2, and Slug) in xenograft tissues derived from TMEM97-deficient MDA-MB-231 cells (Fig. 8F). These results illustrated that TMEM97 knockout inhibited tumor growth in mouse breast cancer xenograft probably through the downregulation of the Wnt/β-catenin signaling pathway.

## Discussion

As a key co-receptor for Wnt/β-catenin signaling, LRP6 is a promising therapeutic target for breast cancer. LRP6 is expressed in breast cancer cells and tissues [32]. Knockdown of LRP6 significantly inhibited Wnt/β-catenin signaling and impaired cell proliferation and tumorigenesis in breast cancer [32]. Multiple small molecular compounds (e.g., salinomycin, prodigiosin, silibinin, rottlerin, gigantol, and CDDO-Me) have been shown to inhibit the Wnt/β-catenin pathway and growth in breast cancer cells by acting on LRP6 [24, 33–37]. It has been reported that the recombinant Mesd protein and its C-terminal region peptide, two antagonists of LRP6, markedly suppressed Wnt/β-catenin signaling, cell proliferation and tumor growth in breast cancer [38]. Overexpression of LRP6 in the mammary glands of mice initiated tumor formation [39]. Moreover, the tumor metastasis suppressor N-myc downstream regulated gene-1 (NDRG1) repressed Wnt/β-catenin signaling through interacting with LRP6, resulting in suppression of metastatic phenotypes of mammary tumor cells [40]. Zhang et al showed that kallistatin, a plasma protein, antagonized the Wnt/β-catenin signaling cascade via binding to LRP6 in human breast cancer MDA-MB-231 cells [41]. Iberahim et al reported that syndecan-1 potentiated Wnt signaling and modulated the cancer stem cell phenotype via the regulation of LRP6 expression in triple-negative breast cancer cells [42]. Sox9 has been shown to mediate the Wnt/β-catenin activation through the regulation of LRP6 and TCF4 expression in breast cancer [43]. These studies indicated that multiple factors may participate in the modulation of the Wnt/β-catenin pathway via targeting LRP6 in breast cancer. However, molecular mechanism of action of these factors in the regulation of LRP6-mediated Wnt signaling cascade remains unclear. In the present study, TMEM97 was identified as a novel LRP6-binding protein. The binding of TMEM97 to LRP6 facilitated the recruitment of CK1δ/ε to LRP6 complex, resulting in LRP6 phosphorylation at Ser1490 and the activation of Wnt signaling. Knockout of TMEM97 abrogated its oncogenic properties through downregulating LRP6-mediated Wnt signaling in breast cancer. Therefore, our results define TMEM97 for the first time as a novel positive regulator of canonical Wnt signaling. We further demonstrated that TMEM97-mediated Wnt signaling plays an important role in the tumorigenesis of breast cancer.

LRP6 plays a crucial role in the initial events of canonical Wnt signaling [44]. When Wnt ligand is absent, the extracellular domain of LRP6 exhibited an autoinhibitory effect on the downstream Wnt signaling cascade. This autoinhibitory effect will be abrogated upon Wnt stimulation [5]. A truncated LRP6 lacking the extracellular domain could constitutively activate Wnt/β-catenin signaling [45]. LRP6 contains a proline-serine rich intracellular region. This region appears to be natively unstructured since proline residues may restrict its conformational space. A conformational change will occur to this region upon Wnt binding to Fzd and LRP6, allowing CK1 to easily access the S/T cluster for phosphorylation [30]. A previous study showed that transmembrane protein 198 (TMEM198) could specifically activate LRP6-mediated Wnt signaling. TMEM198 interacted with LRP6 and recruited CK1 family proteins to phosphorylate serine or threonine residues important for LRP6 activation [46]. TMEM198 is a membrane scaffold protein with 360 amino acids. Topology prediction suggests that TMEM198 consists of a very short extracellular domain, seven transmembrane domains, and a cytoplasmic tail with about 110 amino acids [46]. TMEM97 is a conserved integral membrane protein with 176 amino acids, which is predicted to have a four-pass transmembrane topology. There is no significant sequence identity between TMEM198 and TMEM97. Similar to TMEM198, TMEM97 could interact with LRP6 intracellular domain, potentiate the association of LRP6 with CK1δ/ε and initiate LRP6 phosphorylation. Future studies are needed to determine whether both TMEM198 and TMEM97 potentiate LRP6-mediated Wnt signaling cascade using a common mechanism.

TMEM97 has been implicated in cholesterol and lipid metabolism. This protein has been shown to bind Niemann-Pick C1 (NPC1) protein, a crucial modulator of LDL cholesterol transport out of lysosomes. TMEM97 could form a complex with progesterone receptor membrane component 1 (PGRMC1) and low-density lipoprotein receptor (LDLR), and this trimeric complex was necessary for the rapid internalization and trafficking of LDL [47]. TMEM97 knockdown reduced cellular cholesterol levels as well as the rate of internalization of LDL [48]. We examined the effect of PGRMC1 on TMEM97/LRP6-mediated Wnt signaling. Our results showed that PGRMC1 dose-dependently attenuated the transcription of SuperTOPFlash luciferase reporter activated by TMEM97/LRP6 (Fig. S5). We speculate that PGRMC1 could antagonize Wnt signaling possibly by competing with LRP6 for binding to TMEM97.

TMEM97 was recently identified as a sigma-2 receptor using a chemical biology approach. Alon et al showed that the biological and molecular properties of TMEM97 were identical to that of the sigma-2 receptor. Downregulation of TMEM97 expression by siRNA reduced the binding of the sigma-2 receptor to ^3^H DTG, a sigma-2 receptor ligand. Expression of TMEM97 in cells lacking the sigma-2 receptor conferred a similar sigma-2 receptor binding profile. Furthermore, the Asp29 and Asp56 residues in TMEM97 were identified as ligand binding sites [49]. Zeng et al examined the cytotoxic effects of a panel of structurally diverse sigma-2 ligands, including siramesine, SW43, PB28, RHM1, RHM4, and ISO1. Knockout of TMEM97 in HeLa cells did not affect the EC_50_ values of these sigma-2 ligands, suggesting sigma-2 ligand-induced cytotoxicity is not mediated by TMEM97 [50]. We evaluated the effect of several sigma-2 receptor ligands (glycerol phenylbutyrate, DTG, and siramesine) on TMEM97/LRP6-mediated Wnt signaling. Our results showed that glycerol phenylbutyrate, DTG, and siramesine had little effect on the Wnt signaling pathway activated by TMEM97/LRP6 at the concentrations tested (Fig. S6), suggesting that TMEM97 may enhance the LRP6-mediated signaling cascade in a ligand-independent manner.

## Supplementary information

Supplementary materials
