# Supplementary material for: Transmembrane protein 97 exhibits oncogenic properties via enhancing LRP6-mediated Wnt signaling in breast cancer
Source: Cell Death Dis. 2021 Oct 6;12(10):912. doi: 10.1038/s41419-021-04211-8 (PMC8494741; doi:10.1038/s41419-021-04211-8)
Supplement: Supplementary file 1 — Supplementary materials [file 41419_2021_4211_MOESM1_ESM.docx]

**Supplementary materials-CDDIS-21-1652**

**Supplemental Table S1** Primers used in this study

| **Primer ID** | **Sense (5’-3’)** | **Anti-sense (5’-3’)** |
| --- | --- | --- |
| sgRNA of TMEM97 | CTATGGGGGCTCCGGCAACC |  |
| TMEM97 gene exon 1 | CAAGGGCCAGTGCAGTTTC | TCGCGGGAAACCTTAAACCC |
| AXIN2 | CAACACCAGGCGGAACGAA | GCCCAATAAGGAGTGTAAGGACT |
| LEF1 | AGAACACCCCGATGACGGA | GGCATCATTATGTACCCGGAAT |
| Survivin | AGGACCACCGCATCTCTACAT | AAGTCTGGCTCGTTCTCAGTG |
| Fibronectin | ACCTACGGATGACTCGTGCTTT | TTCAGACATTCGTTCCCACTCA |
| LGR5 | CTCCCAGGTCTGGTGTGTTG | GAGGTCTAGGTAGGAGGTGAAG |
| Sox2 | GCCGAGTGGAAACTTTTGTCG | GGCAGCGTGTACTTATCCTTCT |
| Slug | CGAACTGGACACACATACAGTG | CTGAGGATCTCTGGTTGTGGT |

**Supplemental Table S2** Antibodies used in this study

| **Antibodies** | **Source** | **Category No.** |
| --- | --- | --- |
| Anti-LRP6 antibody | Cell Signaling Technology | 2560 |
| Anti-LRP6 (Ser1490) antibody | Cell Signaling Technology | 2568 |
| Anti-CK1ε antibody | Cell Signaling Technology | 12448 |
| Anti-active β-catenin antibody | Cell Signaling Technology | 8814 |
| Anti-LEF1 antibody | Cell Signaling Technology | 76010 |
| Anti-AXIN2 antibody | Cell Signaling Technology | 2151 |
| Anti-Sox antibody | Cell Signaling Technology | 23064 |
| Anti-Slug antibody | Cell Signaling Technology | 9585 |
| Anti-Flag antibody | Cell Signaling Technology | 14793 |
| Anti-V5 antibody | Cell Signaling Technology | 13202 |
| Anti-CK1δ antibody | Santa Cruz Biotechnology | Sc-55553 |
| Anti-β-catenin antibody | Santa Cruz Biotechnology | Sc-7963 |
| Anti-survivin antibody | Santa Cruz Biotechnology | Sc-17779 |
| Anti-TMEM97 antibody | Novus | NBP1-30436 |
| Anti-LGR5 antibody | Abcam | ab273092 |
| Anti-GAPDH antibody | Proteintech | 60004-1-Ig |
| Anti-α-Tubulin antibody | Proteintech | 66031-1-Ig |
| Anti-His antibody | Proteintech | 10001-0-AP |
| Anti-V5 Affinity Gel | Biotool | B23502 |
| Anti- Flag Affinity Gel | Bimake | B23102 |

**Supplementary Figures**


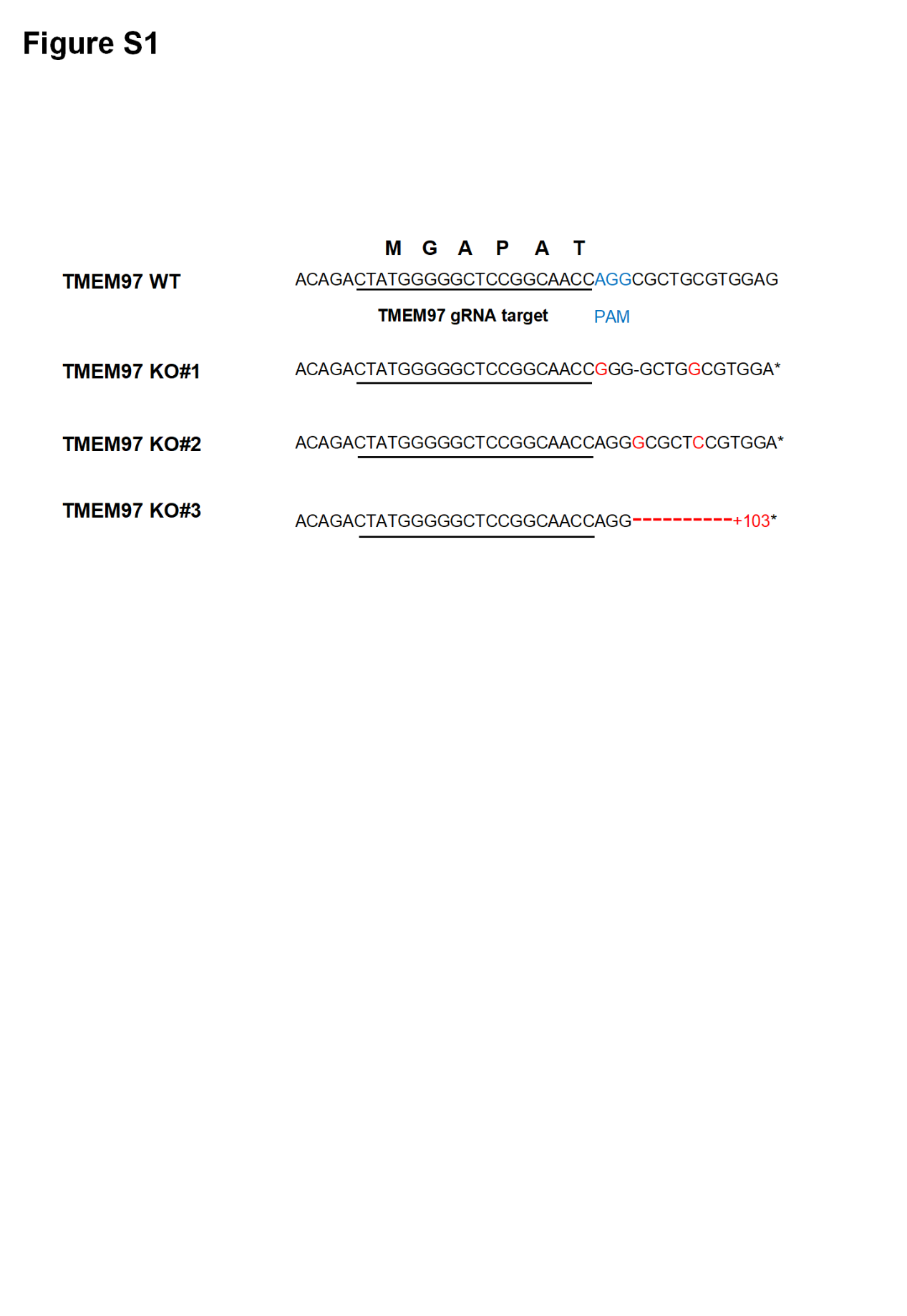


Fig. S1. **DNA sequencing for verifying stable TMEM97 gene knockout in MDA-MB-231 cells.** The target sites were amplified from the extracted DNA of CRISPR-transduced MDA-MB-231 cells, and amplicons were sequenced. The TMEM97 gRNA target sequences are underlined and the PAM sites are indicated in blue. The identified mutations are highlighted in red and frameshift mutations are denoted with an asterisk (*).


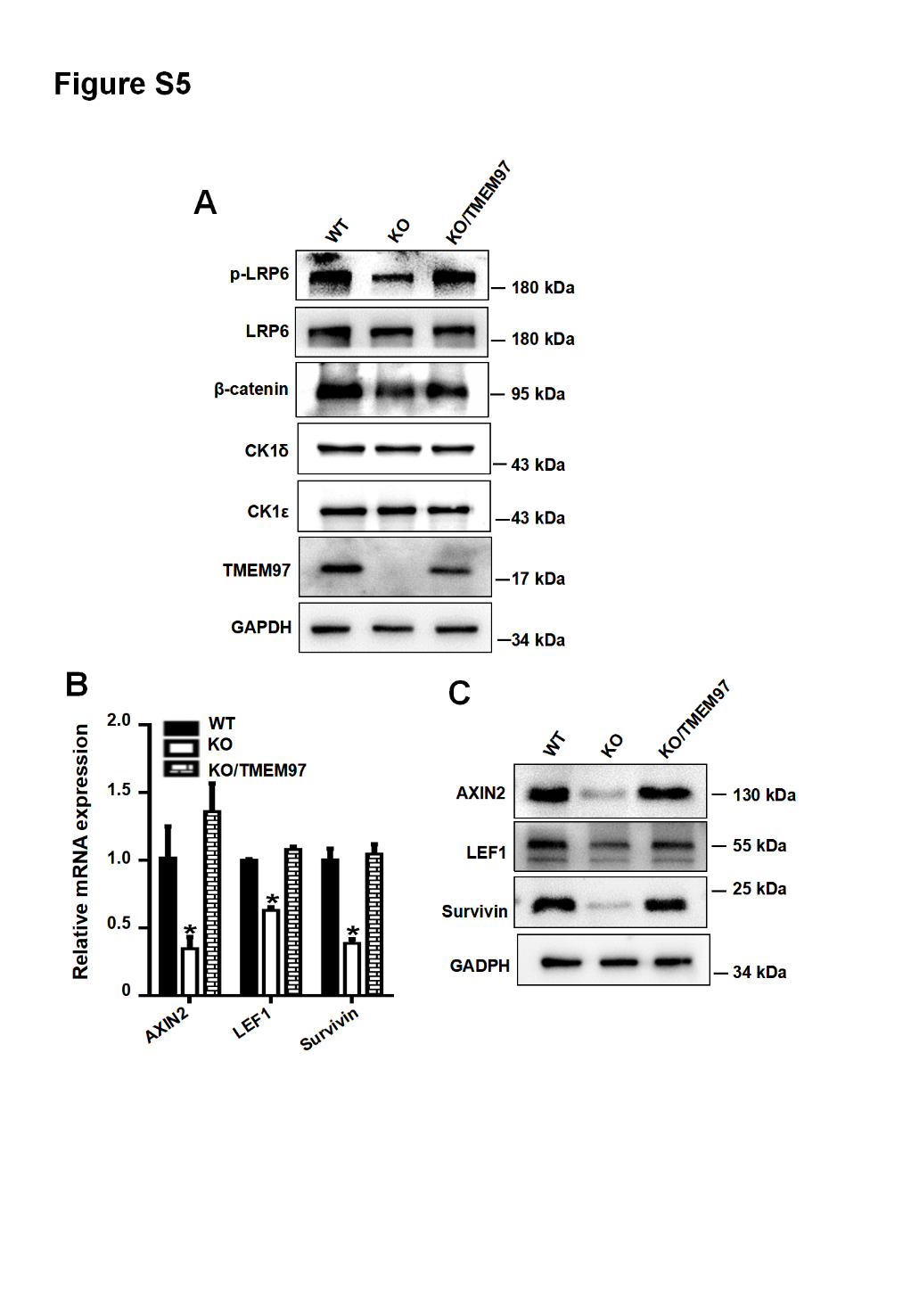


Fig. S2. **TMEM97 reintroduction rescues the effect of TMEM97 knockout on Wnt/β-catenin signaling in MDA-MB-231 cells.** (A) Western blot analysis showing the levels of LRP6, pLRP6, β-catenin, CK1δ, CK1ε and TMEM97 in Parental (WT), TMEM97 knockout (KO) MDA-MB-231 cells and KO cells with TMEM97 reintroduction (KO/TMEM97 cells). GAPDH was used as a loading control. Knockout of TMEM97 downregulated the Wnt/β-catenin pathway, which was reversed by reintroduction of TMEM97 in MDA-MB-231 cells. (B) Real-time PCR analysis of Wnt target genes AXIN2, LEF1, survivin, and fibronectin in WT, KO cells and KO/TMEM97 MDA-MB-231 cells. Data represent the mean ± SD (n = 3, **P* < 0.05). (C) Western blot analysis of the protein expression of Wnt target genes AXIN2, LEF1, and survivin in WT, KO cells and KO/TMEM97 MDA-MB-231 cells. The images shown are representative of data generated in at least three independent experiments.


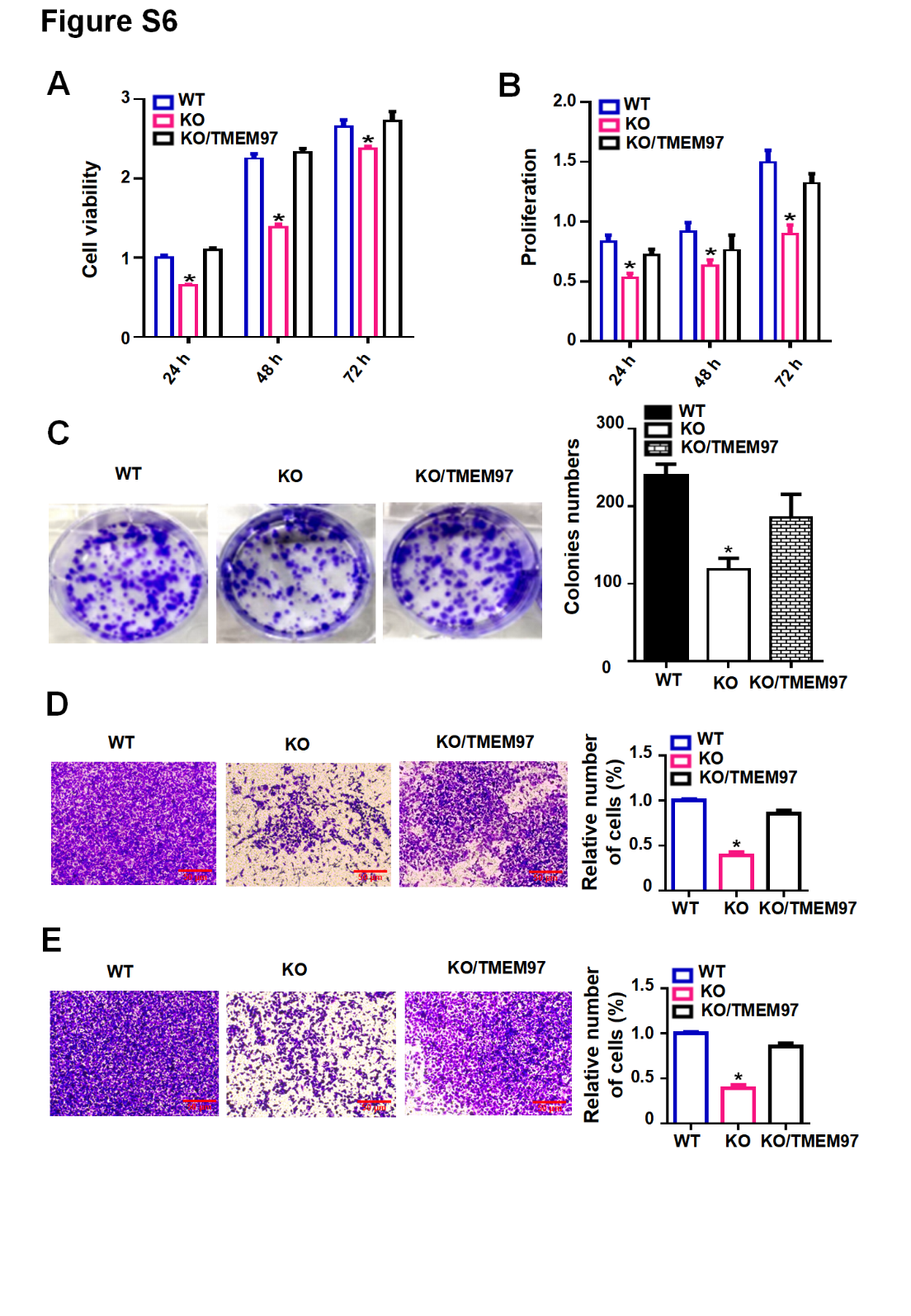


Fig. S3. **TMEM97 reintroduction rescues the effect of TMEM97 deficiency on cell viability, proliferation, colony formation, migration and invasion in MDA-MB-231 cells.** Parental (WT), TMEM97 knockout (KO) MDA-MB-231 cells and KO cells with TMEM97 reintroduction (KO/TMEM97 cells) were seeded at 1×10^3^ cells/well in 96-well plates. After 24 h, 48 h, and 72 h of culture, MTT assay was used to detect cell viability (A). Cell proliferation was detected using BrdU incorporation assay (B). (C) Colony formation of WT, KO cells and KO/TMEM97 MDA-MB-231 cells. The right panel is the diagrammatic representation of quantitative data which showed the relative number of colonies formed in knockout cells and their parental counterparts at day 10. The number of colonies was calculated by the ImageJ software (n = 3). (D, E) Migration and invasion of WT, KO cells and KO/TMEM97 MDA-MB-231 cells. The right panel is the quantitative data on the relative number of migrated or invaded cells in the graphs (n = 3). Scale bar = 500 μm. Data were shown as mean ± SD. *P <0.05 compared with vehicle control.


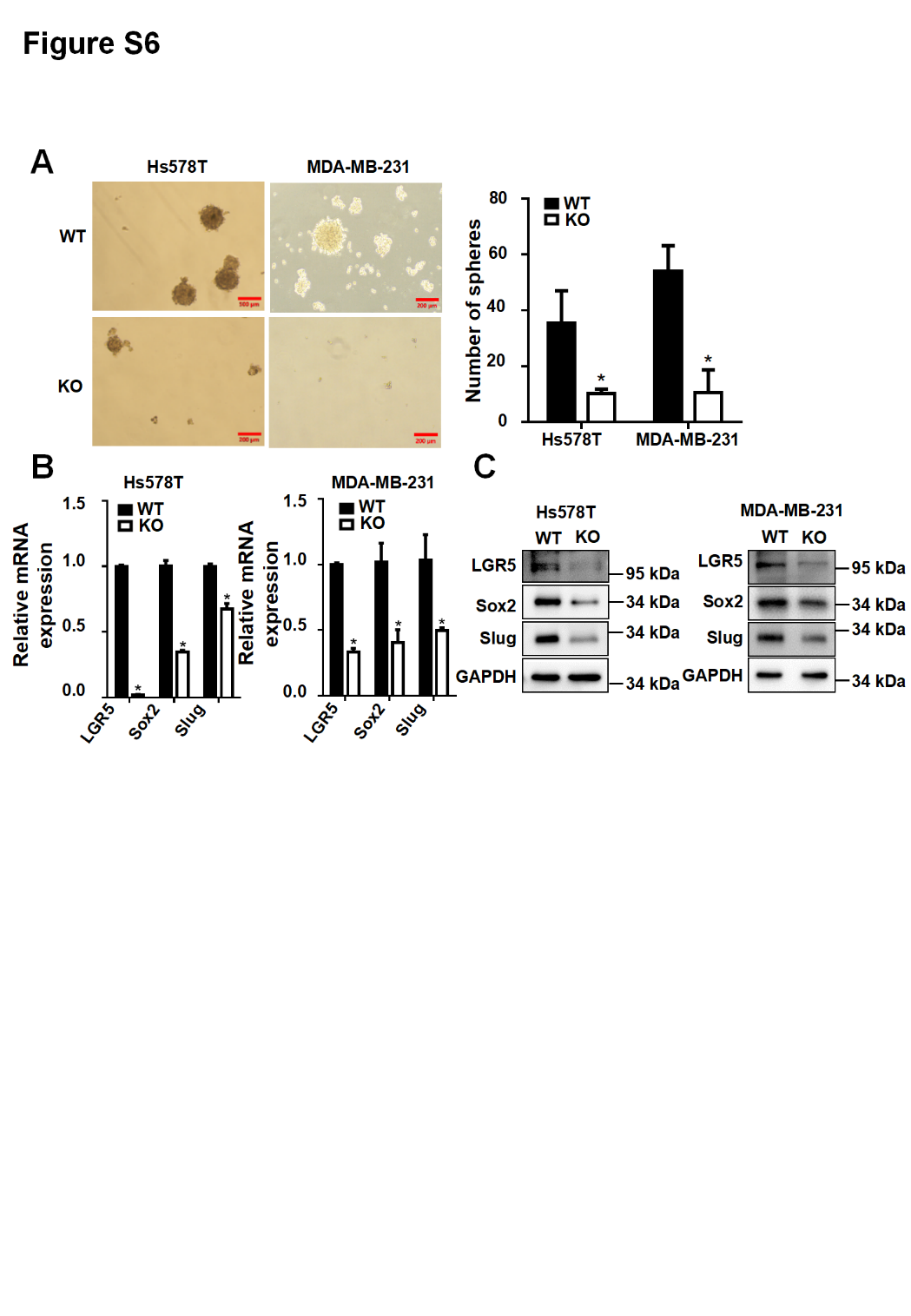


Fig. S4. **TMEM97 knockout inhibits the stemness properties of breast cancer cells.** (A) TMEM97 knockout Hs578T and MDA-MB-231 cells and their parental counterparts were cultured in 24-well ultra-low attachment plates to detect the ability of sphere formation. Scale bar = 200 μm. Diagrammatic representation of quantitative data showed the relative number of spheres formed at day 10. Each cell line had three replicates. **P* <0.05, versus vehicle control; Dunn’s multiple comparisons test. (B) The mRNA levels of stemness marker genes LGR5, Sox2, and Slug were analyzed using real-time PCR in TMEM97 knockout Hs578T and MDA-MB-231 cells and their parental counterparts. (C) The protein levels of stemness marker genes LGR5, Sox2, and Slug were measured by immunoblotting. The images shown are representative of data generated in at least three independent experiments.


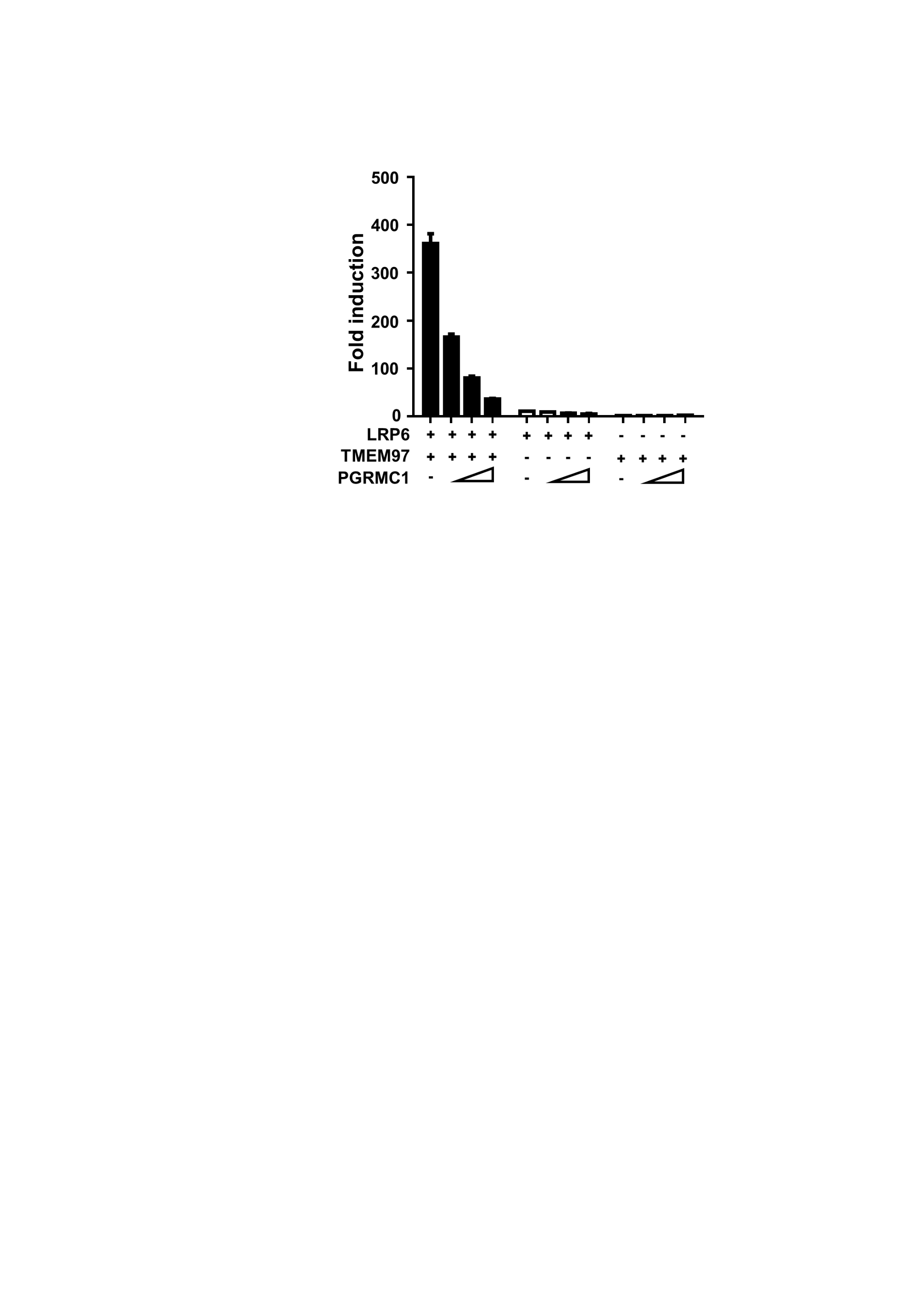


Fig. S5. **PGRMC1 dose-dependently attenuates the Wnt/β-catenin signaling pathway activated by TMEM97/LRP6.** (A) The SuperTOPFlash reporter gene was transfected into HEK293T cells together with expression vectors for TMEM97 and LRP6, in the absence or presence of increasing amounts of PGRMC1 expression plasmid (0, 50, 100, and 200 ng).


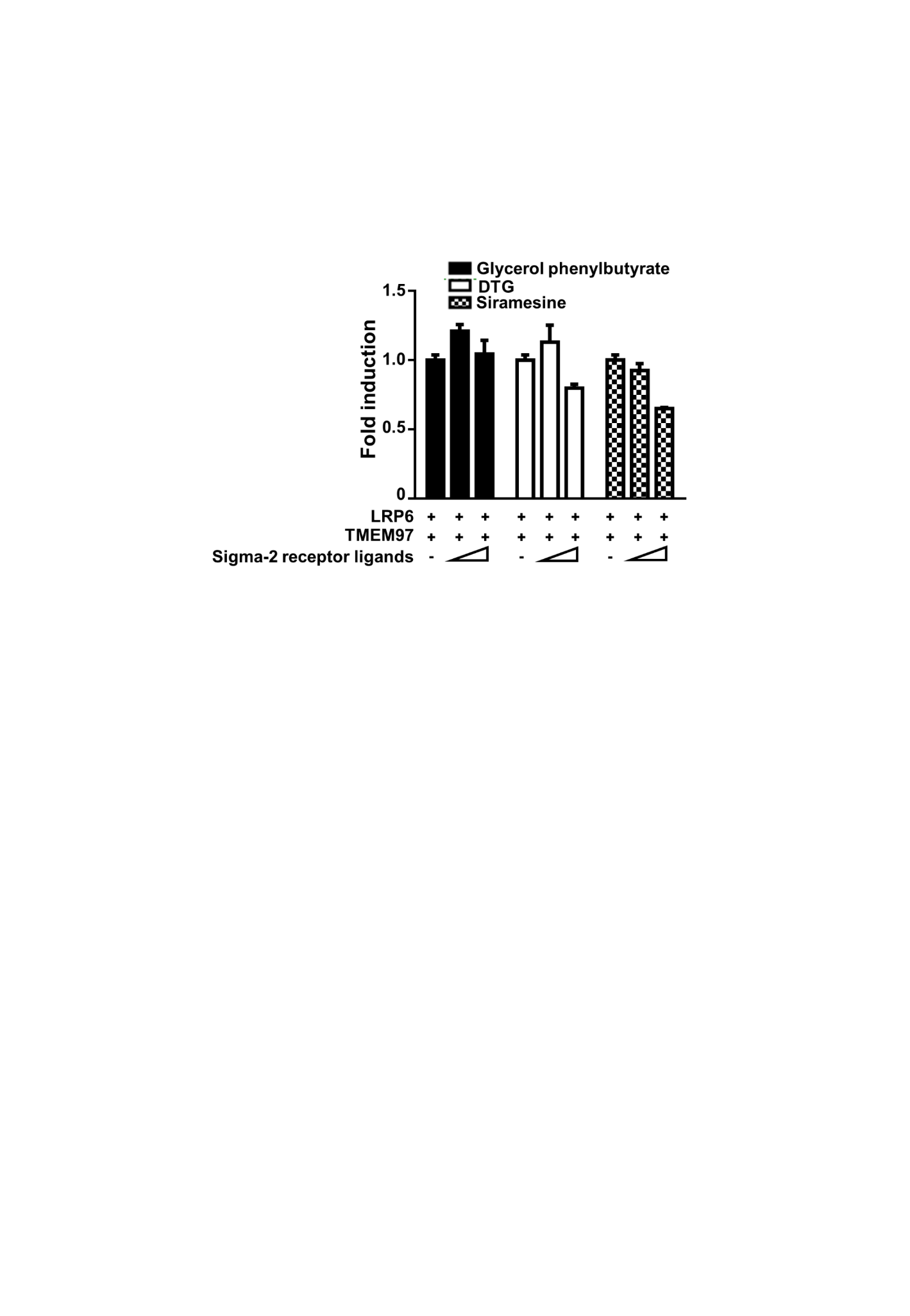


Fig. S6. **Sigma-2 receptor ligands glycerol phenylbutyrate, DTG, and siramesine have little effect on TMEM97/LRP6-mediated Wnt signaling.** (A) The SuperTOPFlash reporter gene was transfected into HEK293T cells together with expression vectors for TMEM97 and LRP6, after which cells were treated with phenylbutyrate, DTG, and siramesine at 1 and 10 μM, respectively.
